# Pan-Cancer Bioinformatics Analysis of Gene UBE2C

**DOI:** 10.3389/fgene.2022.893358

**Published:** 2022-04-27

**Authors:** Lin Yuan, Zhenyu Yang, Jing Zhao, Tao Sun, Chunyu Hu, Zhen Shen, Guanying Yu

**Affiliations:** ^1^ School of Computer Science and Technology, Qilu University of Technology (Shandong Academy of Sciences), Jinan, China; ^2^ School of Computer and Software, Nanyang Institute of Technology, Nanyang, China; ^3^ Department of Gastrointestinal Surgery, Central Hospital Affiliated to Shandong First Medical University, Jinan, China

**Keywords:** gene Expression, pan-cancer, GSE data, prognostic analysis, TCGA, immune infiltration

## Abstract

Ubiquitin-Conjugating Enzyme E2 C (UBE2C) is a gene that encodes protein. Disorders associated with UBE2C include methotrexate-related lymphatic hyperplasia and complement component 7 deficiency. The encoded protein is necessary for the destruction of mitotic cell cyclins and cell cycle progression, and may be involved in cancer progression. In this paper, on the basis of public databases, we study the expression differential mechanism of gene expression of UBE2C in various tumors and the performance of prognosis, clinical features, immunity, methylation, etc.

## Introduction

UBE2C (Ubiquitin-Conjugating Enzyme E2 C) is a gene that encodes protein. Disorders associated with UBE2C include methotrexate-related lymphatic hyperplasia and complement component 7 deficiency. The encoded protein is necessary for the destruction of mitotic cell cyclins and cell cycle progression, and may be involved in cancer progression. Genes play a very critical function in the impact of cancer on the human body. Genes also regulate life activities by influencing the activities of biological factors (e.g., lncRNA, DNA methylation, etc.) (Khan et al., 2020; Yuan et al., 2021a; Yuan et al., 2021b).

In this paper, on the basis of public databases, we study the expression differential mechanism of gene expression of UBE2C in various tumors and the performance of prognosis, clinical features, immunity, methylation, etc (Sayers et al., 2019; Yuan and Huang, 2019; Warwick Vesztrocy and Dessimoz, 2020). Gene UBE2C play a very critical function in the impact of cancer on the human body. As far as we know, there is currently no pan-cancer analysis of UBE2C.

## Methods and Materials

In this section, on the basis of public databases, we study the expression differential mechanism of gene expression of UBE2C in various tumors and the performance of prognosis, clinical features, immunity, methylation, etc (Peng et al., 2018; Yuan et al., 2018; Consortium, 2019).

### Gene Expression Level in Various Tissues

**FIGURE 1:**
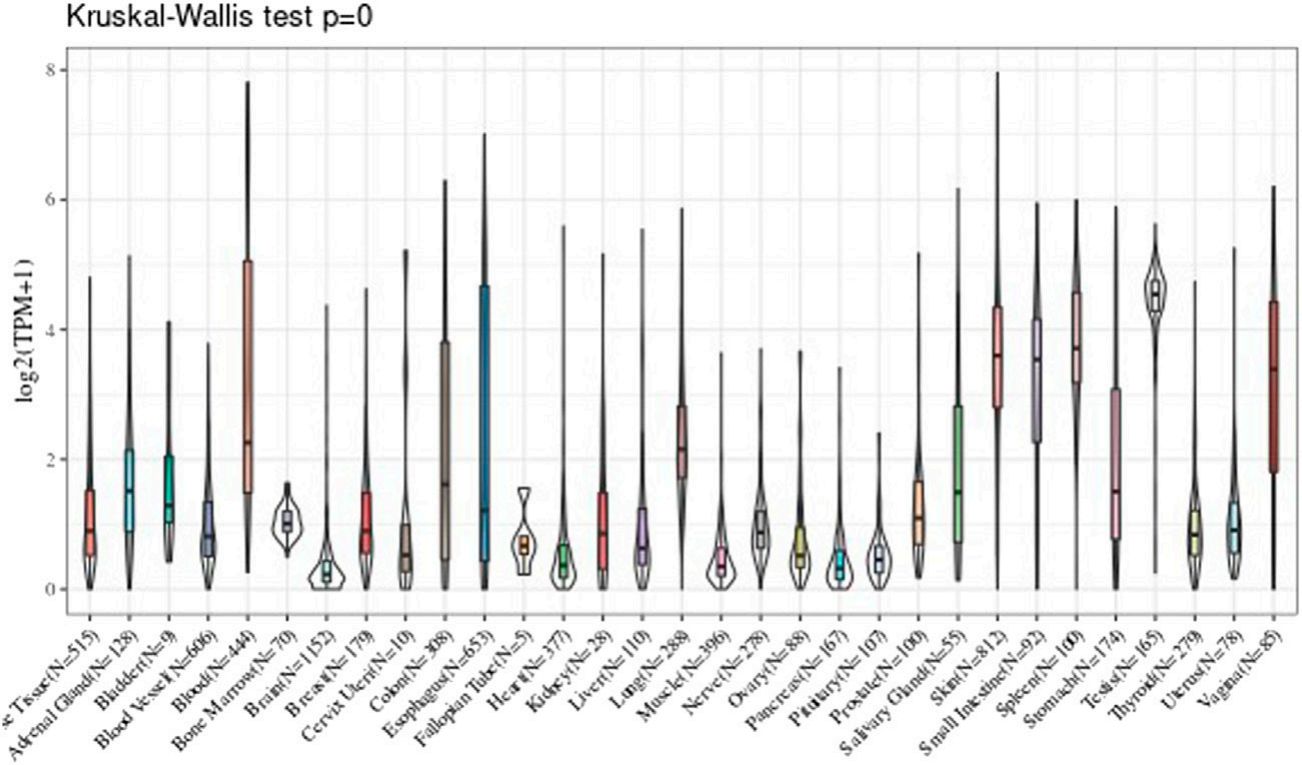
The expression level of UBE2C gene in 31 tissues.

In the part, the GTEx data were used to observe the UBE2C gene expression level in those various tissues, and we observed the gene expression in 31 tissues. The results are shown in Figure 1.

### Tumor Tissues Analysis

**FIGURE 2:**
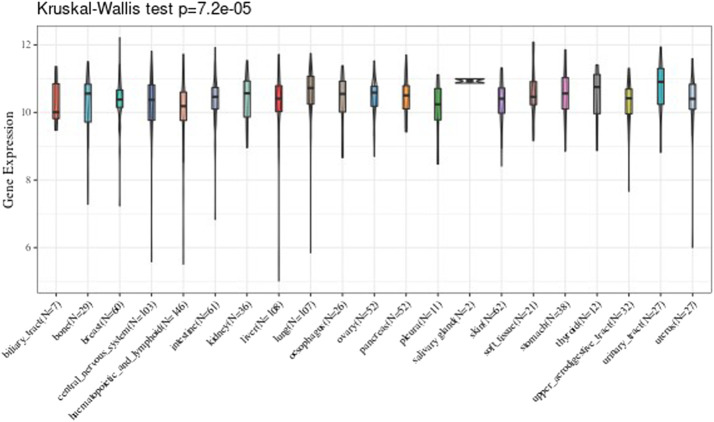
Comparison of the expression value level of UBE2C gene in twenty-one tissues.

We analyzed every cell line of tumor downloaded from the CCLE, and analyzed the 21 tissues expression level according to the source of the tissue. The results are shown in Figure 2.

### Analysis of Tumor Data and Non-Tumordata From TCGA

**FIGURE 3:**
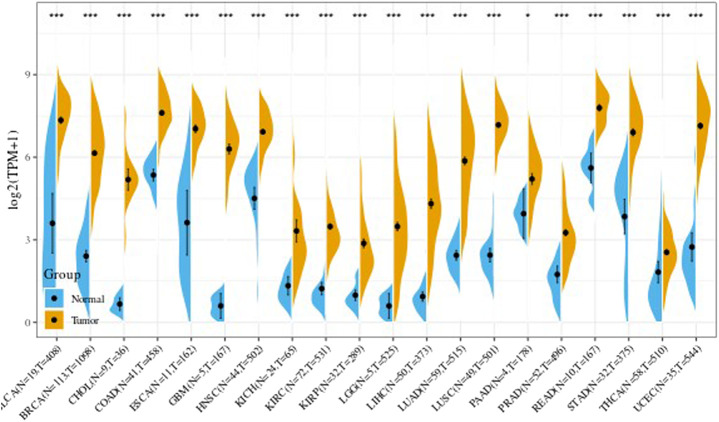
The expression level of UBE2C gene in TCGA tumor and nontumor.

We further obtained from the TCGA database the difference in the UBE2C gene expression in every tumor sample between tumor and adjacent tumor. Results are shown in Figure 3. In the figure, * indicates *p* less than 0.05, ** indicates *p* < 0.01, and *** indicates *p* < 0.001.

### Joint Analysis of GTEx and TCGA Data

Due to the small number of normal samples in TCGA, we found the data from the GTEx database, and integrated these data with the data in TCGA tumor tissues to analyze the differences in expression value of 27 tumors. The results are shown in the Supplementary Figure S1.

### Prognostic Analysis of Gene Ubiquitin-Conjugating Enzyme E2 C in Pan-Cancer

**FIGURE 4:**
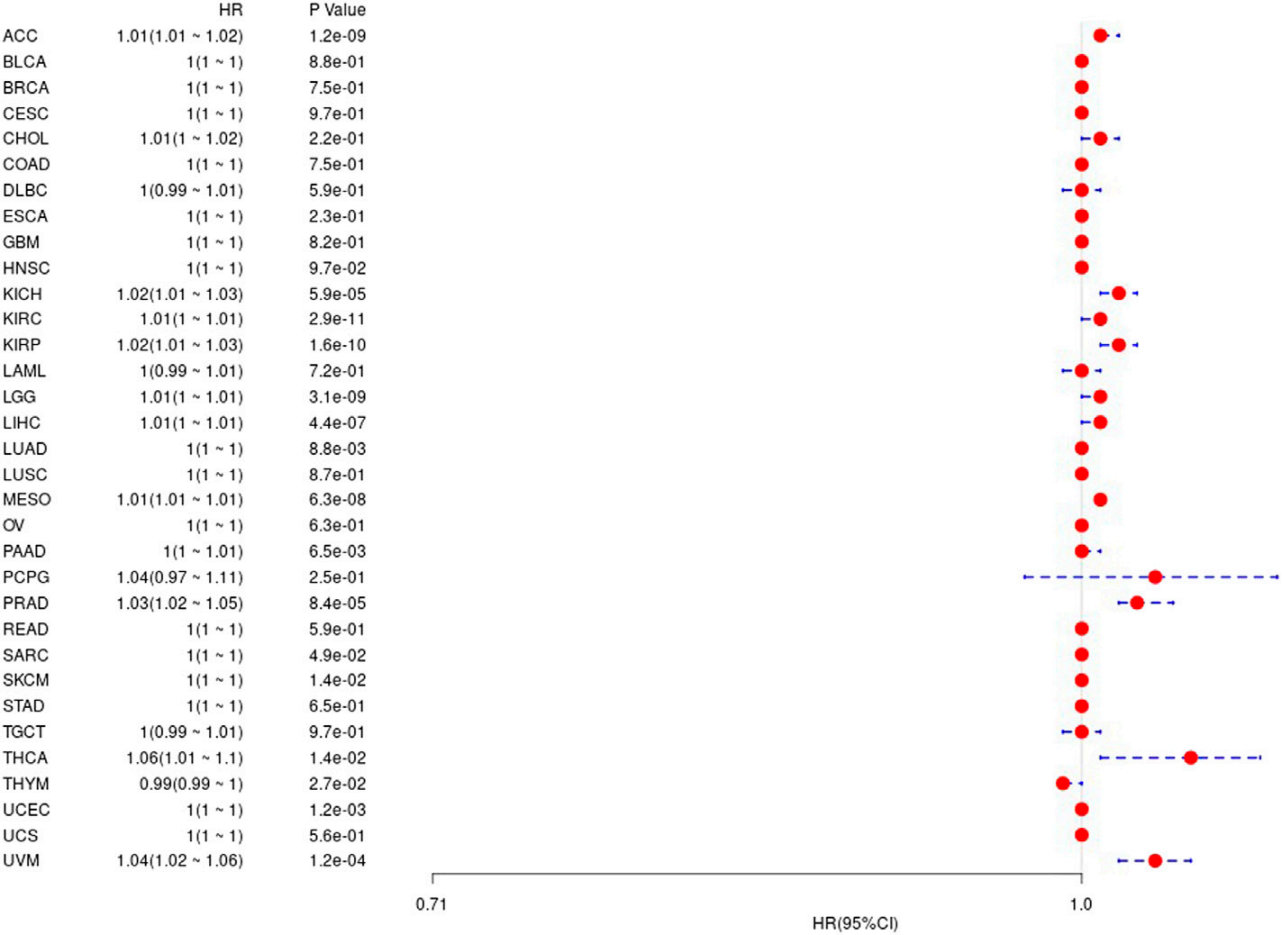
The relationship between UBE2C gene and overall survival time in days in TCGA tumor and nontumor.

We first used the data of gene expression to analyze the difference between expression and prognosis in thirty-three tumors of TCGA. Using single-factor survival analysis, the forest plot of the gene UBE2C we studied in 33 tumors is shown in Figure 4. The prognostic K-M curve results are shown in Supplementary Figure S2.

**FIGURE 5:**
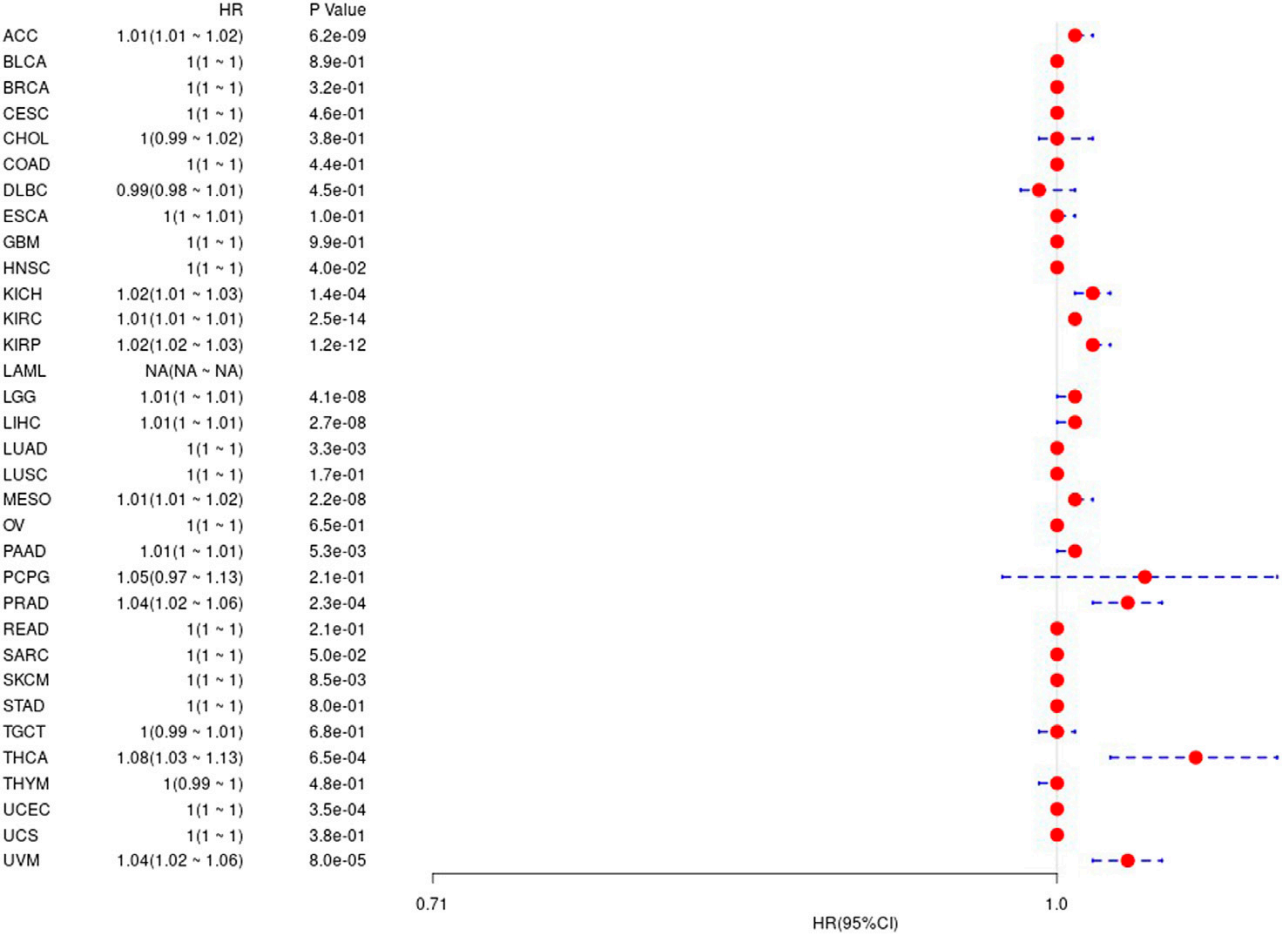
The relationship between UBE2C gene and disease-specific survival in TCGA tumor and nontumor.

We considered the non-tumor death factors that may exist during patient follow-up, and we analyzed the difference between gene expression values and prognosis in 33 tumors in TCGA, and the results are shown in the Supplementary Figure S2. The corresponding prognostic K-M curve results are shown in Figure 5.

**FIGURE 6:**
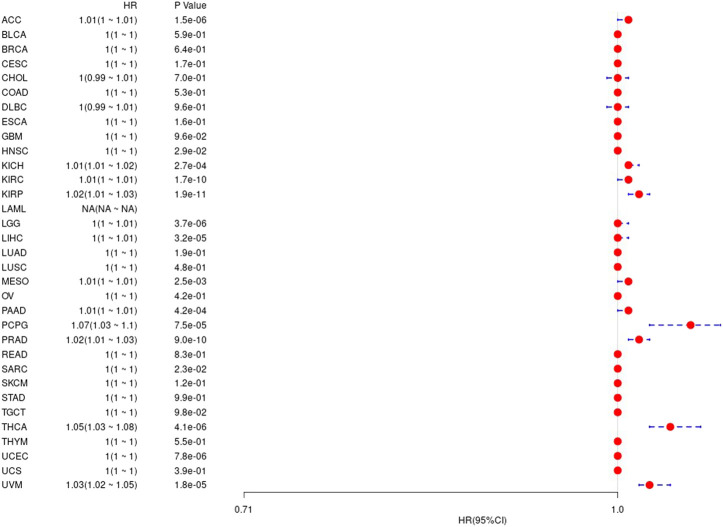
The relationship between UBE2C gene and disease-free interval in TCGA tumor and nontumor.

We further analyzed difference between gene value and prognosis in 33 TCGA tumors, and the results are shown in Supplementary Figure S3 and Figure 6, respectively.

**FIGURE 7:**
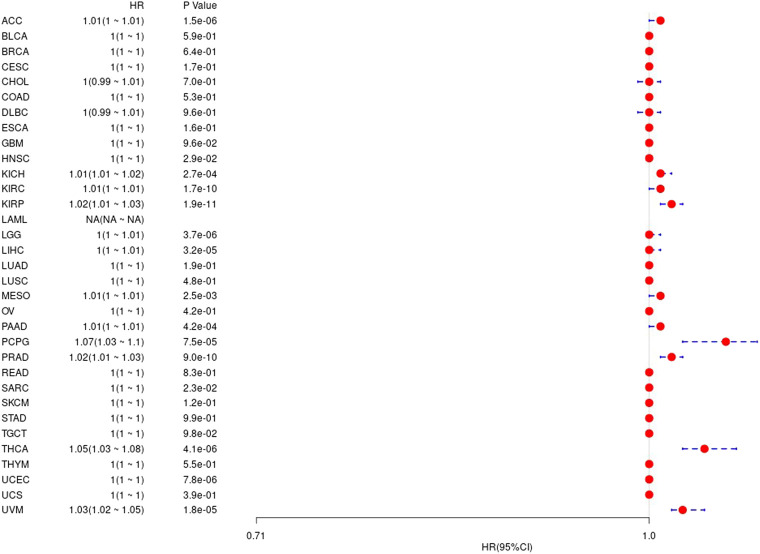
The relationship between UBE2C gene and prognosis-free interval in TCGA tumor and nontumor.

At the same time, we also calculated the difference between gene expression value and prognosis-free interval in 33 TCGA tumors. The results are shown in Supplementary Figure S4 and Figure 7.

### Correlation Between Genes and Immune Infiltration Value

Tumor infiltrating lymphocytes (TIL) are an independent predictor of the status and survival of the sentinel lymph node in cancer (Yuan et al., 2017; Peng et al., 2018). We studied the association between the gene expression values and the values of these cells of immune. We studied the correlation of each tumor (e.g., ACC, BRCA, CODA, LUAD, etc.), the three most significantly related tumors are shown in the Supplementary Figure S5.

### Study on Immune Scoring of Gene Expression

**FIGURE 8:**
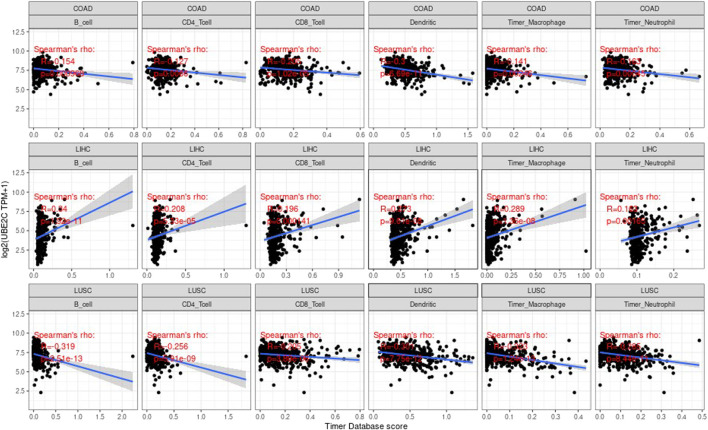
The most significant expression of the top 3 three tumor immune infiltration levels.

More and more papers have shown that the tumor immune play a very critical function in the impact of cancer on the human body. The R packages named estimate was used to calculate the immune scores and stromal scores of each tumor sample, and observed gene values and immunity in thirty-three tumors. The score is like ImmuneScore, and the association between gene expression value and matrix score is like StromalScore (Yuan et al., 2016; Liu et al., 2018; Simion et al., 2019). In the immune score of gene expression ESTIMATE, the first three tumors that are most significantly related are shown in the Figure 8.

### The Correlation Between Gene Value and Immune Checkpoint Genes

**FIGURE 9:**
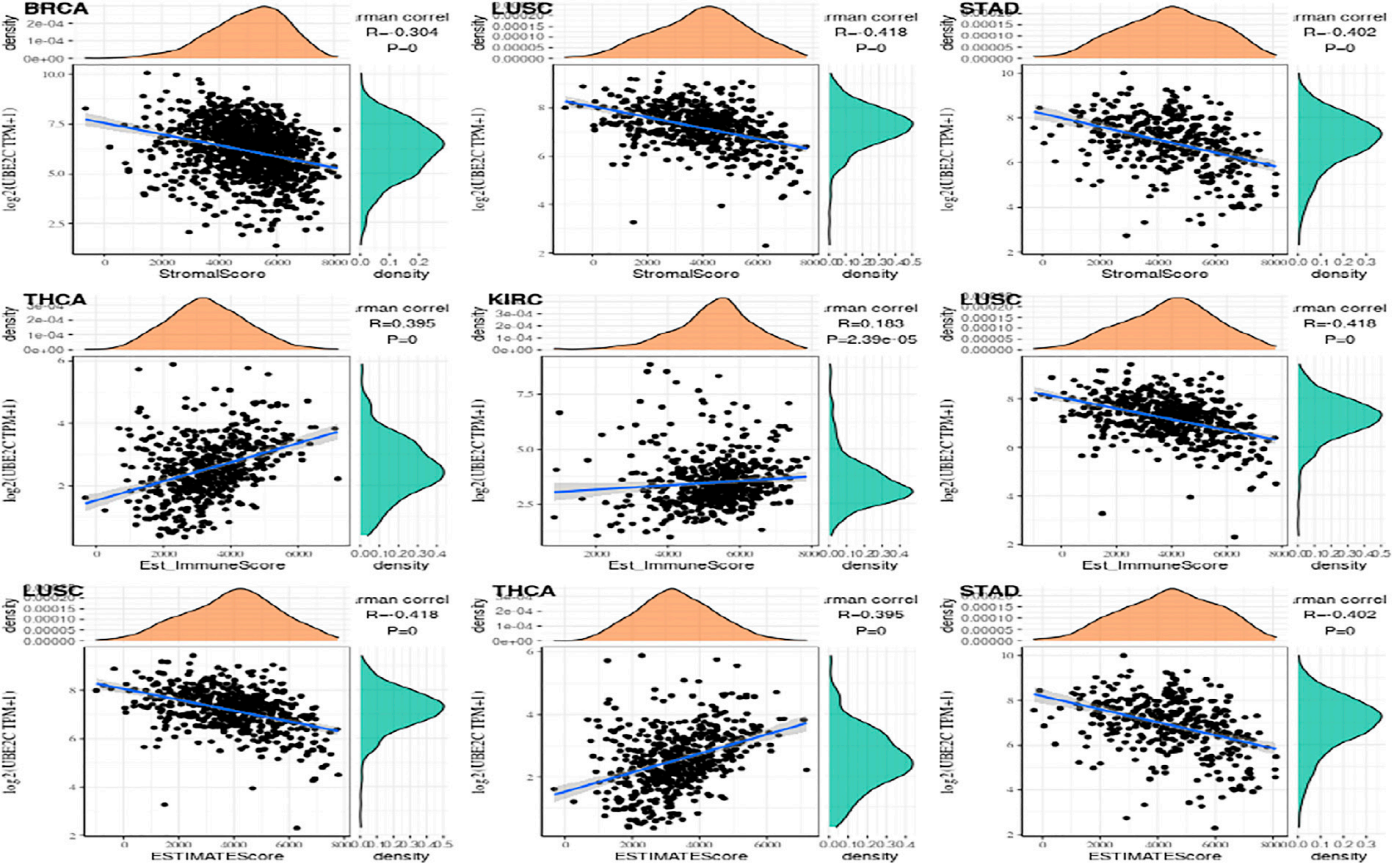
The top 3 tumors that are most significantly related.

Under normal circumstances, the immune system can recognize and eliminate tumor cells through a series of biochemical reactions. However, tumor cells can adopt different strategies to suppress the body’s immune system and escape being eliminated. And prevent tumor cells from killing tumor cells normally. Each stage of the response has survived. Tumor immunotherapy can control and eliminate tumors by restarting and activating tumor immune circulation to restore the body’s normal anti-tumor immune response (Wei et al., 2016; Guan et al., 2018). Including monoclonal antibody, therapeutic antibodies, cancer vaccines, and small molecule inhibitor (Yuan et al., 2015; Ge et al., 2017). Based on the relevant database, we collected more than 40 common immune check-related genes, and then we calculated and analyzed the relationship between the gene value and the immune checkpoint gene value. We calculated the immune checkpoint gene value separately and calculated the relationship with the target gene The correlation of the expressed value is as follows. In the figure, * indicates a significant relevance value *p* less than 0.05, ** indicates a significant relevance value *p* less than 0.01, *** indicates a significant relevance value *p* < 0.001. The results are shown in Figure 9.

### The Relationship Between Gene Value and Immune Neoantigens

Tumor neoantigens are neoantigens encoded by mutated genes of tumor cells. They are mainly generated by gene point mutations, deletion mutations, gene fusions, etc., which are new and abnormal proteins that are different from those expressed by normal cells. The polypeptide fragments formed by enzymatic hydrolysis of these proteins are presented to T cells as antigens by DC cells. Based on these antigens, T cells are transformed into mature activated T cells that recognize tumor neoantigens. These antigens can increase the number of these activated T cells. Using the immune activity of neoantigens, we can design and synthesize neoantigen vaccines according to the mutations of tumor cells, and then immunize patients to achieve therapeutic effects (Zheng et al., 2014; Le et al., 2017). Here we separately count the amount of neoantigens in every sample from tumor and analyze the differnece between gene expression and the amount of antigens is shown in Supplementary Figure S6.

### Association Between Gene Value and Neoantigen

**FIGURE 10:**
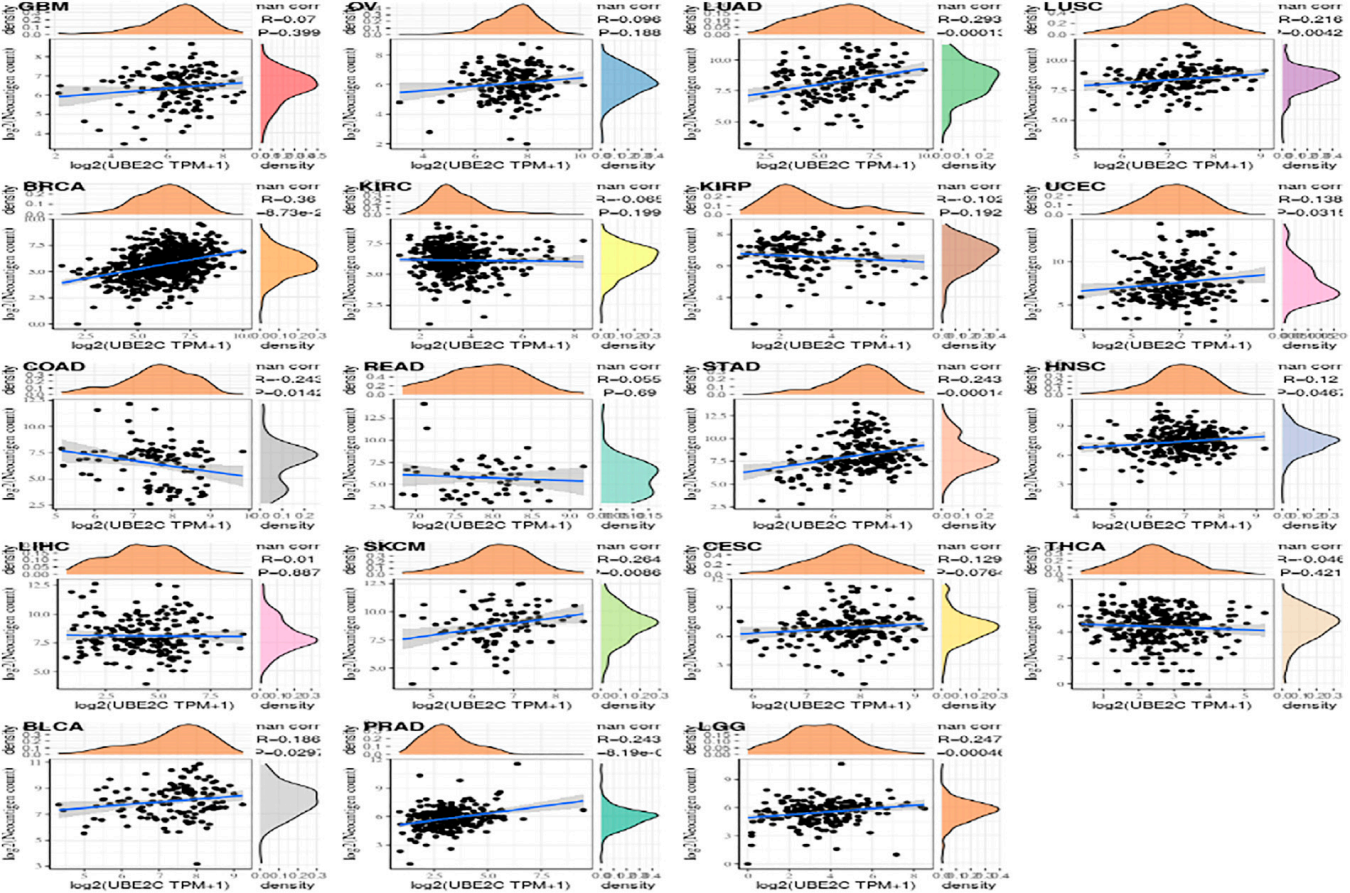
The relationship between gene expression and the number of antigens.

We measure the mutation load of the tumor by counting the number of somatic mutations in the coding region of the tumor cell genome on an average of 1 Mb. Sometimes it is also directly expressed by the total amount of non-synonymous mutations. The types of mutations mainly contain mononuclear mutations. A lot of mutations such as SNV and insertion/deletion of small fragments. TMB is often used to show the amount of mutations contained in tumor sample cells and is a measurable biomarker (Ju et al., 2019). Here we separately count the TMB of every tumor sample, and analyze the association between value of gene expression and TMB as shown in Figure 10, where we use Spearman rank correlation coefficient.

### The Association Between Gene Value and Neoantigen

**FIGURE 11:**
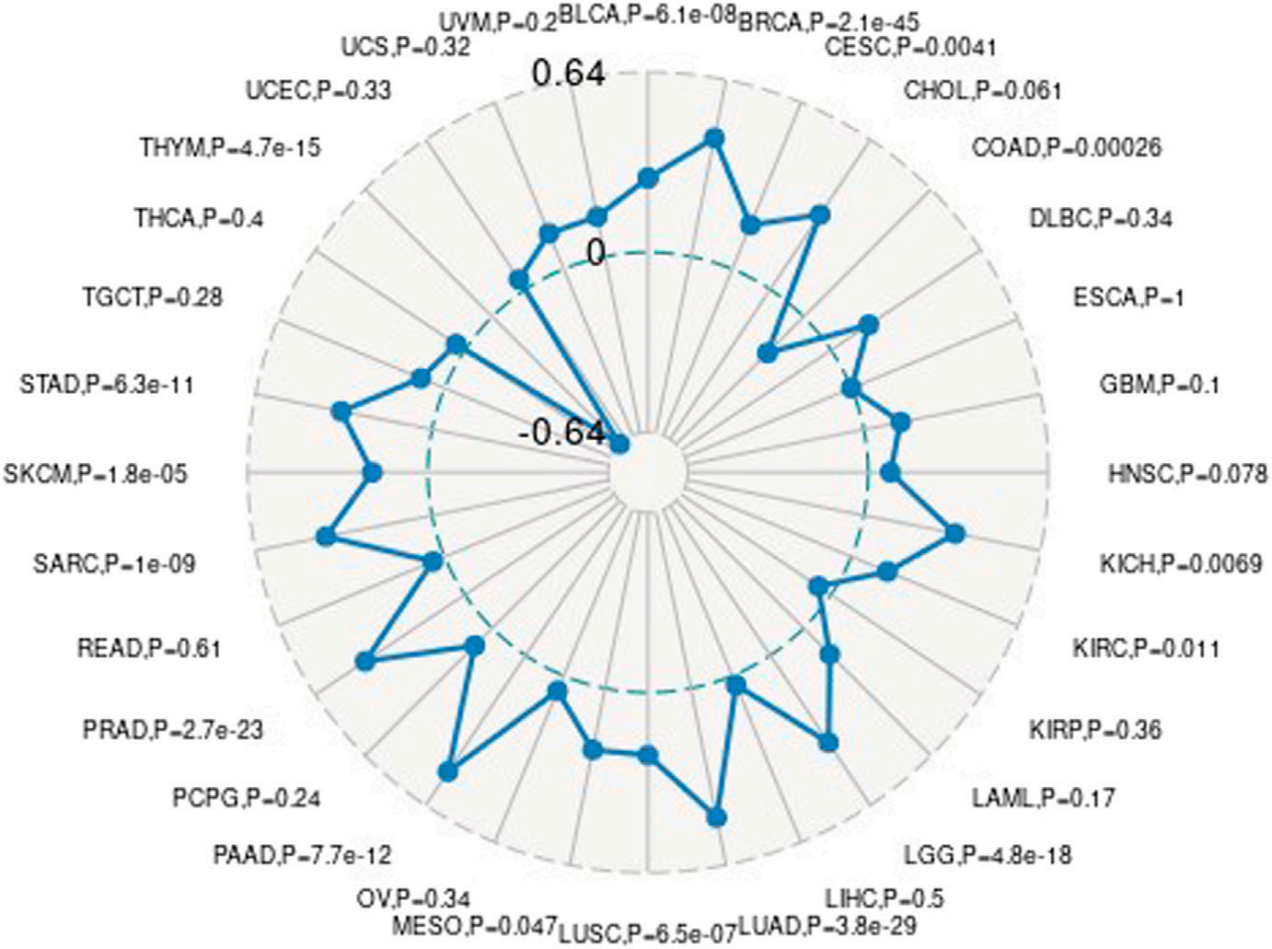
The relationship between gene expression and TMB.

Microsatellite instability refers to any variation in the extent of a microsatellite caused by the insertion of a repeat unit in a tumor compared with normal tissues, and the appearance of new microsatellite alleles. We calculated the association between value of gene expression and microsatellite instability as shown in the Figure 11. Among them, we use Spearman’s rank correlation coefficient.

### Gene Mutation Patterns in Various Tumor Samples

We downloaded the variation data of 33 cancers processed by mutect from TCGA, and calculated the mutations of genes in these cancers (e.g., ACC, BLCA, BRCA, etc.). The schematic diagram of the tumors with the most variation is as follows. The results are shown in Supplementary Figure S7.

### The Association Between Gene Expression Value in Each Tumor Sample and Expression Value of Repair Genes and Methyltransferase

**FIGURE 12:**
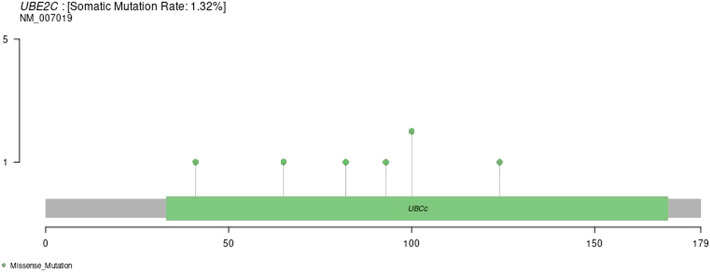
Gene variation data in 33 tumors.

DNA methylation is an important form of DNA modification. DNA methylation can change genetic performance without altering the DNA sequence. DNA methylation can lead to changes in DNA conformation, chromatin structure, DNA stability, and the way in which DNA interacts with proteins, thereby controlling value of genes. Under the action of DNA methylation transferase, DNA methylation binds a methyl group to the covalent bond of the genomic CpG dinucleotide. Then we can calculate the association between value of genes and the expression of four methyltransferases, as shown in Figure 12.

### GSEA Analysis of the Expression of Genes in a Certain Tumor

**FIGURE 13:**
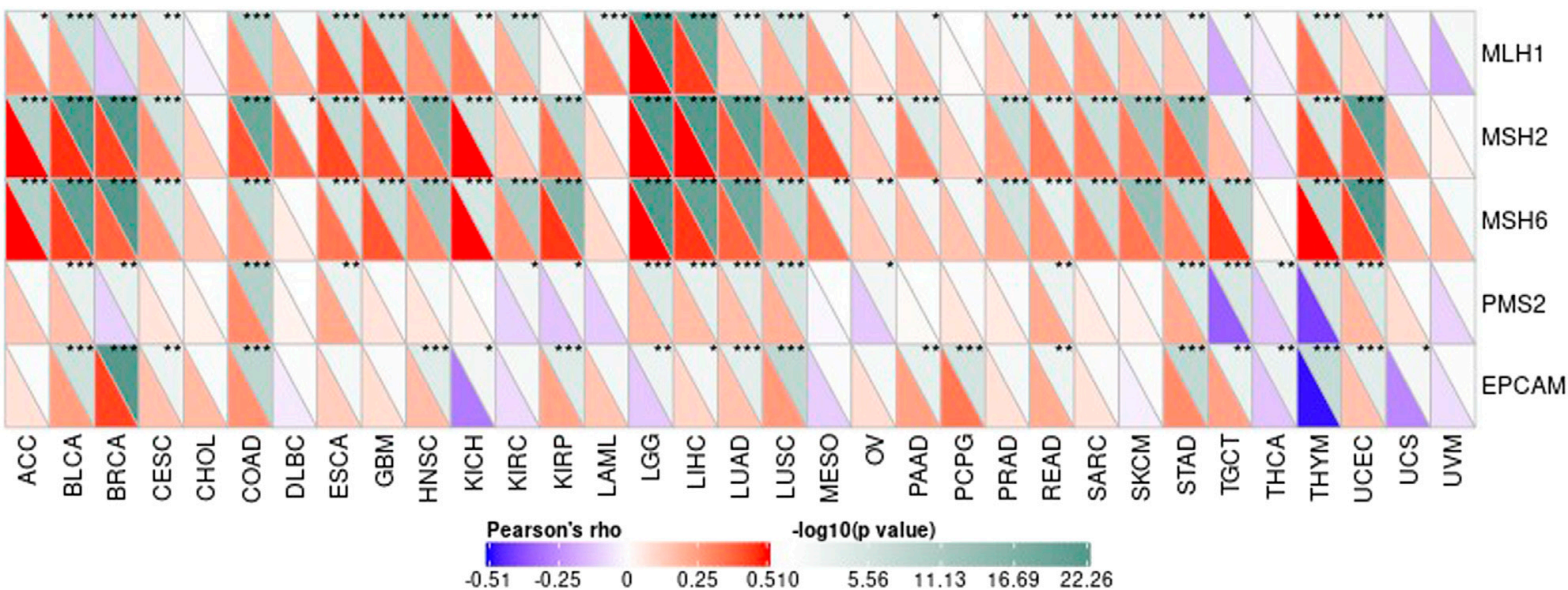
The association between value of genes and the expression of four methyltransferases.

**FIGURE 14:**
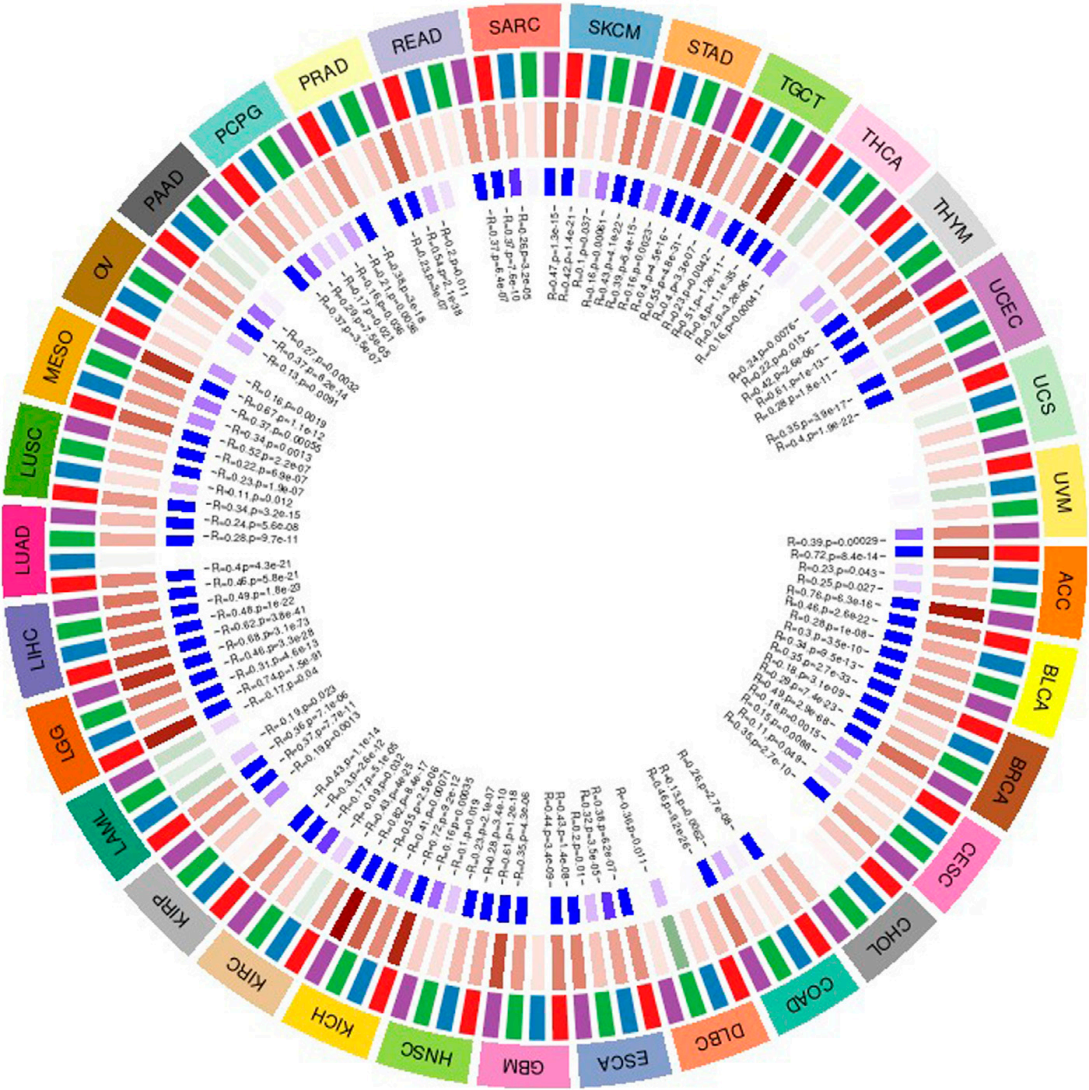
The association between value of genes and the expression of four methyltransferases.

**FIGURE 15:**
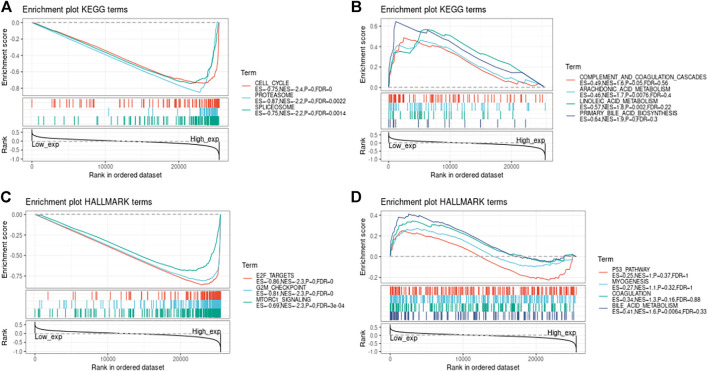
Enrichment performance of genes in KEGG and HALLMARK pathways.

In order to observe the impact of gene on cancers, we divided the cancer samples into high expression and low expression groups according to value of gene expression, and used GSEA to calculate the enrichment of the KEGG and HALLMARK pathways in the high value of gene expression combined low expression group. Results are shown in the Figures 13, 14, 15.

## Conclusion and Discussion

In this article, we proposed to perform pan-cancer expression analysis, pan-cancer prognosis analysis, immune relationship analysis in tumors, gene and immune neoantigens, TMB, and microsatellite instability relationship analysis, and study the mutation patterns of genes in various tumor samples. The relationship between DNA modification and methyltransferase expression is analyzed. The KEGG and HALLMARK enrichment performance of genes are analyzed. Gene UBE2C has a strong correlation with tumors and needs further verification in future clinical trials. Recent studies have shown that UBE2C is associated with many cancers (Zhang et al., 2018; Chiang et al., 2020; Wang et al., 2021). The data used in the paper comes from public databases GEO and TCGA. However, this study has several limitations. First, *in vivo* and *in vitro* experiments are required to further validate our findings. Second, the interaction mechanism between genes is very complex, we need to further study the relationship between UBE2C and other genes.

## Data Availability

The original contributions presented in the study are included in the article/Supplementary Material, further inquiries can be directed to the corresponding author.

## Supplementary Material

Supplementary Figure S1 The gene level of UBE2C in tumor and normal data of TCGA and GTEx.

Supplementary Figure S2 The K-M curve.

Supplementary Figure S3 The corresponding prognostic K-M curve.

Supplementary Figure S4 The corresponding prognostic K-M curve.

Supplementary Figure S5 The corresponding prognostic K-M curve.

Supplementary Figure S6 Relationship between gene expression and immune checkpoint gene expression.

Supplementary Figure S7 The relationship between gene expression and MSI.

## References

[B1] Chiang A.-J. Li C.-J. Tsui K.-H. Chang C. Chang Y.-c. I. Chen L.-W. (2020). UBE2C Drives Human Cervical Cancer Progression and Is Positively Modulated by mTOR. Biomolecules 11 (1), 37. 10.3390/biom11010037 33396624 PMC7823929

[B2] Consortium G. O. (2019). The Gene Ontology Resource: 20 Years and Still GOing strong. Nucleic Acids Res. 47 (D1), D330–D338. 10.1093/nar/gky1055 30395331 PMC6323945

[B3] Ge S. G. Xia J. Sha W. Zheng C. H. (2017). Cancer Subtype Discovery Based on Integrative Model of Multigenomic Data. Ieee/acm Trans. Comput. Biol. Bioinform 14 (5), 1115–1121. 10.1109/TCBB.2016.2621769 28113782

[B4] Guan R. Wang X. Yang M. Q. Zhang Y. Zhou F. Yang C. (2018). Multi-label Deep Learning for Gene Function Annotation in Cancer Pathways. Scientific Rep. 8 (1), 1–9. 10.1038/s41598-017-17842-9 PMC5762767 29321535

[B5] Ju Y. Yuan L. Yang Y. Zhao H. (2019). CircSLNN: Identifying RBP-Binding Sites on circRNAs via Sequence Labeling Neural Networks. Front. Genet. 10, 1184. 10.3389/fgene.2019.01184 31824574 PMC6886371

[B6] Khan H. Reale M. Ullah H. Sureda A. Tejada S. Wang Y. (2020). Anti-cancer Effects of Polyphenols via Targeting P53 Signaling Pathway: Updates and Future Directions. Biotechnol. Adv. 38, 107385. 10.1016/j.biotechadv.2019.04.007 31004736

[B7] Le T. D. Zhang J. Liu L. Li J. (2017). Computational Methods for Identifying miRNA Sponge Interactions. Brief Bioinform 18 (4), 577–590. 10.1093/bib/bbw042 27273287

[B8] Liu J. Lichtenberg T. Hoadley K. A. Poisson L. M. Lazar A. J. Cherniack A. D. (2018). An Integrated TCGA Pan-Cancer Clinical Data Resource to Drive High-Quality Survival Outcome Analytics. Cell 173 (2), 400–e11. e11. 10.1016/j.cell.2018.02.052 29625055 PMC6066282

[B9] Peng J. Zhang X. Hui W. Lu J. Li Q. Liu S. (2018). Improving the Measurement of Semantic Similarity by Combining Gene Ontology and Co-functional Network: a Random Walk Based Approach. BMC Syst. Biol. 12 (2), 18–116. 10.1186/s12918-018-0539-0 29560823 PMC5861498

[B10] Sayers E. W. Cavanaugh M. Clark K. Ostell J. Pruitt K. D. Karsch-Mizrachi I. (2019). GenBank. Nucleic Acids Res. 47 (D1), D94–D99. 10.1093/nar/gky989 30365038 PMC6323954

[B11] Simion V. Haemmig S. Feinberg M. W. (2019). LncRNAs in Vascular Biology and Disease. Vasc. Pharmacol. 114, 145–156. 10.1016/j.vph.2018.01.003 PMC6078824 29425892

[B12] Wang Y. Wang J. Tang Q. Ren G. (2021). Identification of UBE2C as Hub Gene in Driving Prostate Cancer by Integrated Bioinformatics Analysis. PloS one 16 (2), e0247827. 10.1371/journal.pone.0247827 33630978 PMC7906463

[B13] Warwick Vesztrocy A. Dessimoz C. (2020). Benchmarking Gene Ontology Function Predictions Using Negative Annotations. Bioinformatics 36 (Suppl. ment_1), i210–i218. 10.1093/bioinformatics/btaa466 32657372 PMC7355306

[B14] Wei P. J. Zhang D. Xia J. Zheng C. H. (2016). LNDriver: Identifying Driver Genes by Integrating Mutation and Expression Data Based on Gene-Gene Interaction Network. BMC bioinformatics 17 (17), 467. 10.1186/s12859-016-1332-y 28155630 PMC5259866

[B15] Yuan L. Huang D. S. (2019). A Network-Guided Association Mapping Approach from DNA Methylation to Disease. Sci. Rep. 9 (1), 5601–5616. 10.1038/s41598-019-42010-6 30944378 PMC6447594

[B16] Yuan L. Zheng C.-H. Xia J.-F. Huang D.-S. (2015). Module Based Differential Coexpression Analysis Method for Type 2 Diabetes. Biomed. Research International 2015, 1–8. 10.1155/2015/836929 PMC4538423 26339648

[B17] Yuan L. Zhu L. Guo W. L. Zhou X. Zhang Y. Huang Z. (2016). Nonconvex Penalty Based Low-Rank Representation and Sparse Regression for eQTL Mapping. Ieee/acm Trans. Comput. Biol. Bioinform 14 (5), 1154–1164. 10.1109/TCBB.2016.2609420 28114074

[B18] Yuan L. Yuan C.-A. Huang D.-S. (2017). FAACOSE: A Fast Adaptive Ant colony Optimization Algorithm for Detecting SNP Epistasis. Complexity 2017, 1–10. 10.1155/2017/5024867

[B19] Yuan L. Guo L. H. Yuan C. A. Zhang Y. H. Han K. Nandi A. (2018). Integration of Multi-Omics Data for Gene Regulatory Network Inference and Application to Breast Cancer. Ieee/acm Trans. Comput. Biol. Bioinform 16 (3), 782–791. 10.1109/TCBB.2018.2866836 30137012

[B20] Yuan L. Zhao J. Sun T. Shen Z. (2021). A Machine Learning Framework that Integrates Multi-Omics Data Predicts Cancer-Related LncRNAs. BMC bioinformatics 22 (1), 1–18. 10.1186/s12859-021-04256-8 34134612 PMC8210375

[B21] Yuan L. Sun T. Zhao J. Shen Z. (2021). A Novel Computational Framework to Predict Disease-Related Copy Number Variations by Integrating Multiple Data Sources. Front. Genet. 12, 696956. 10.3389/fgene.2021.696956 34267783 PMC8276077

[B22] Zhang H. Q. Zhao G. Ke B. Ma G. Liu G. L. Liang H. (2018). Overexpression of UBE2C Correlates with Poor Prognosis in Gastric Cancer Patients. Eur. Rev. Med. Pharmacol. Sci. 22 (6), 1665–1671. 10.26355/eurrev_201803_14578 29630110

[B23] Zheng C. H. Yuan L. Sha W. Sun Z. L. (2014). Gene Differential Coexpression Analysis Based on Biweight Correlation and Maximum Clique. BMC Bioinformatics 15 (15), S3–S7. 10.1186/1471-2105-15-S15-S3 PMC4271563 25474074

