# Supplementary figures and images for: Pan-Cancer Bioinformatics Analysis of Gene UBE2C
Source: Front Genet. 2022 Apr 27;13:893358. doi: 10.3389/fgene.2022.893358 (PMC9091452; doi:10.3389/fgene.2022.893358)

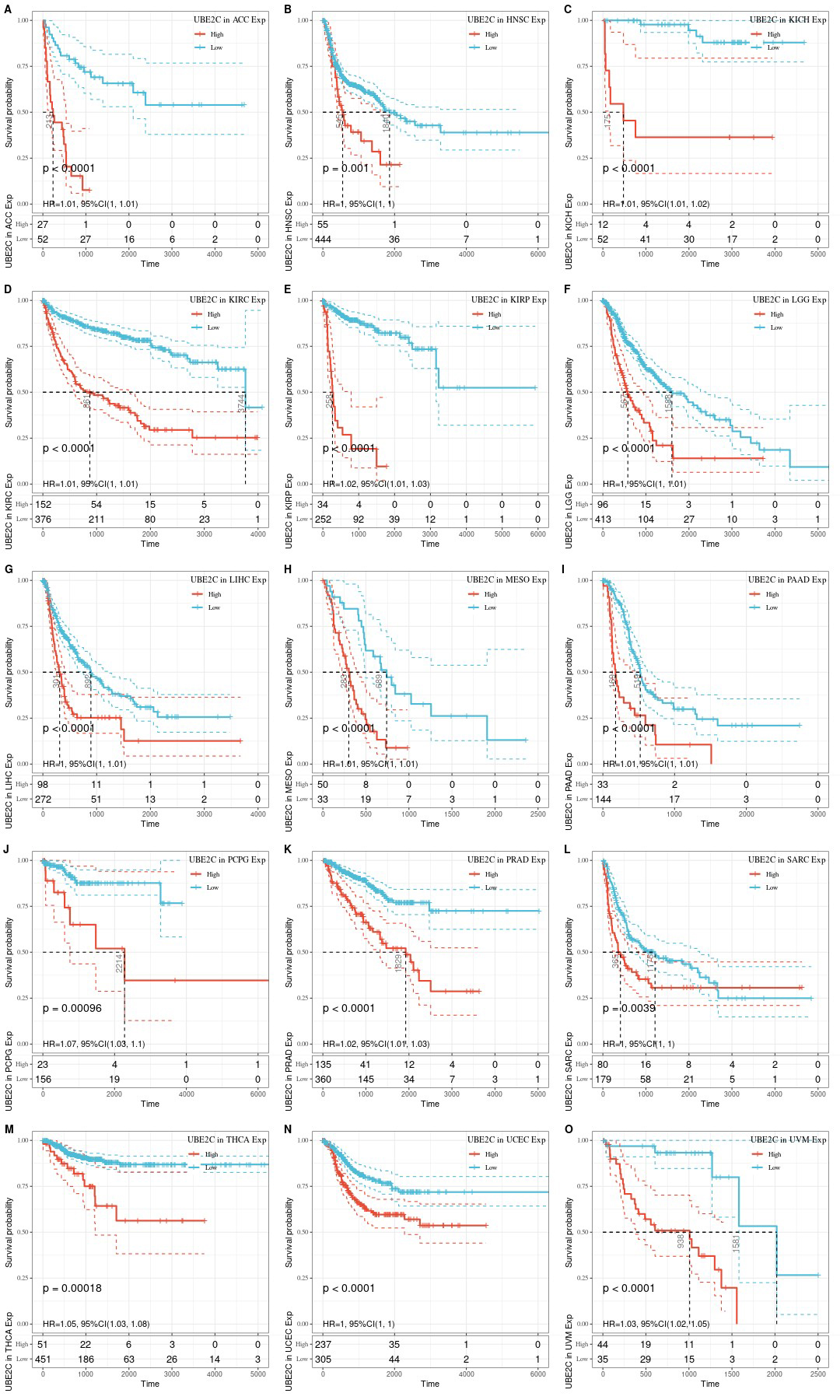

Supplement: Supplementary file 1 [file Image5.jpg]

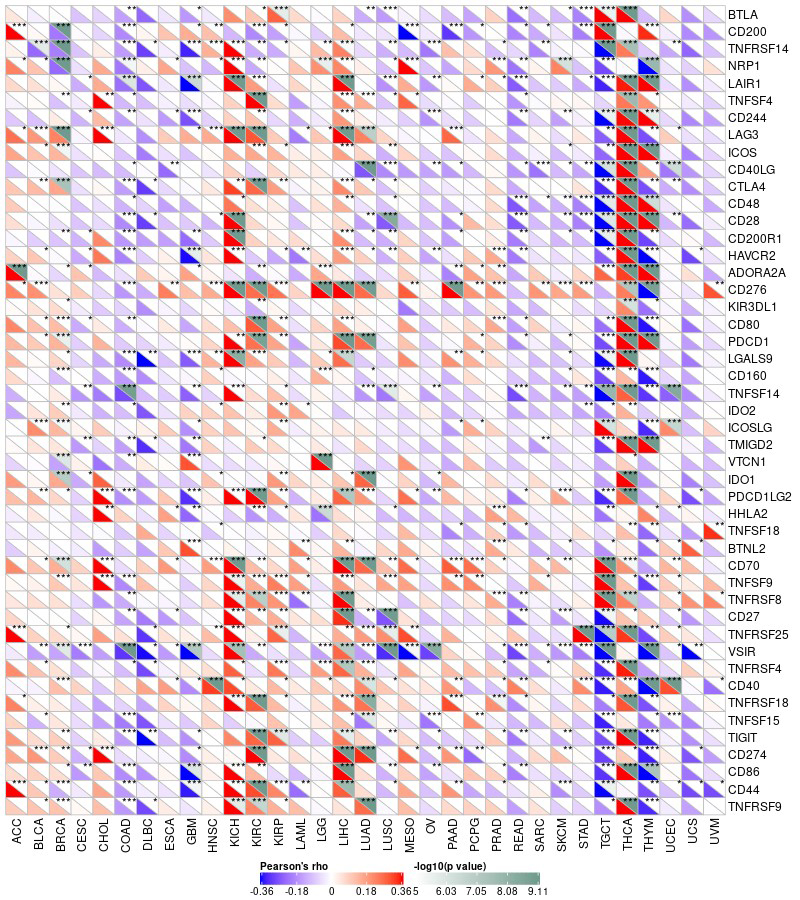

Supplement: Supplementary file 2 [file Image6.jpg]

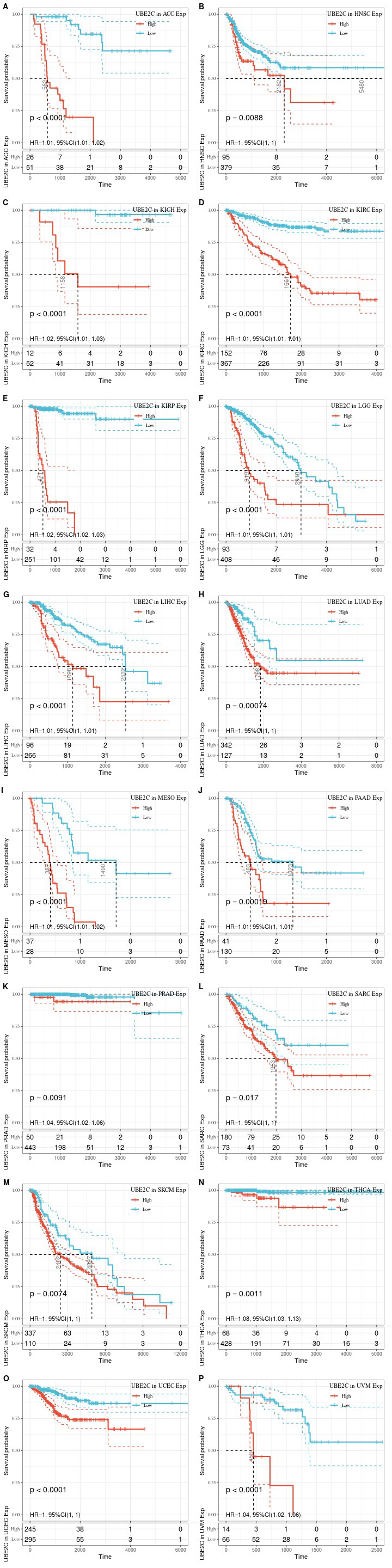

Supplement: Supplementary file 3 [file Image3.jpg]

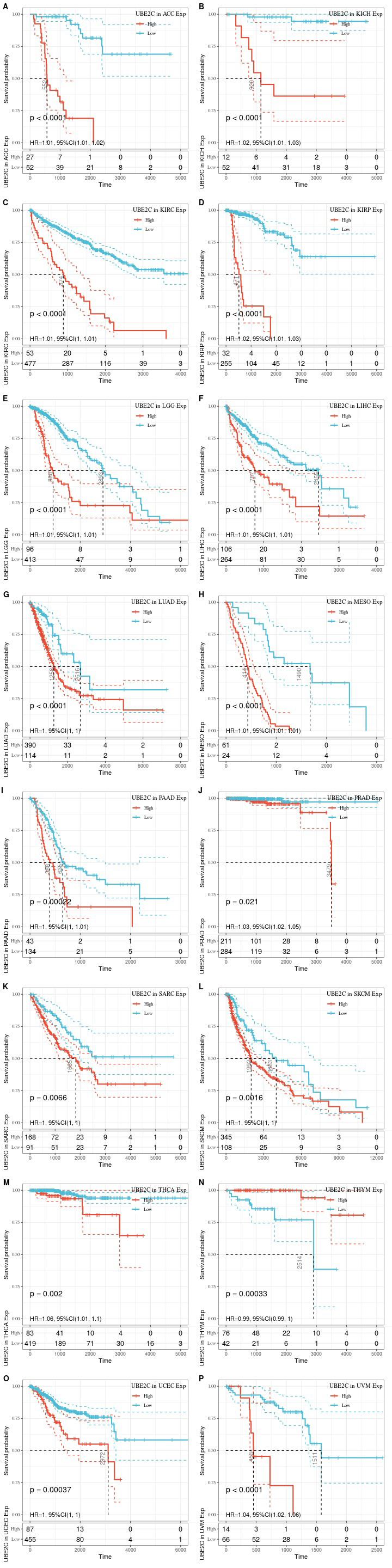

Supplement: Supplementary file 4 [file Image2.jpg]

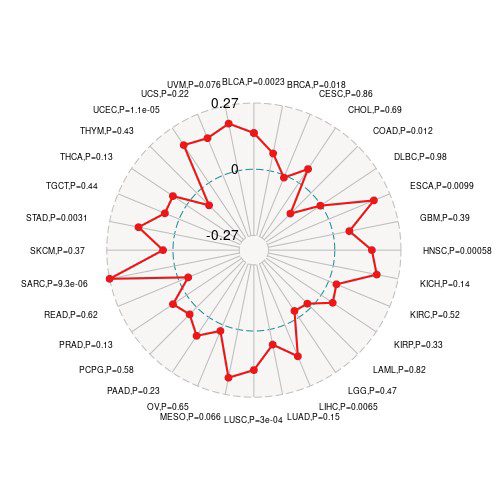

Supplement: Supplementary file 5 [file Image7.jpg]

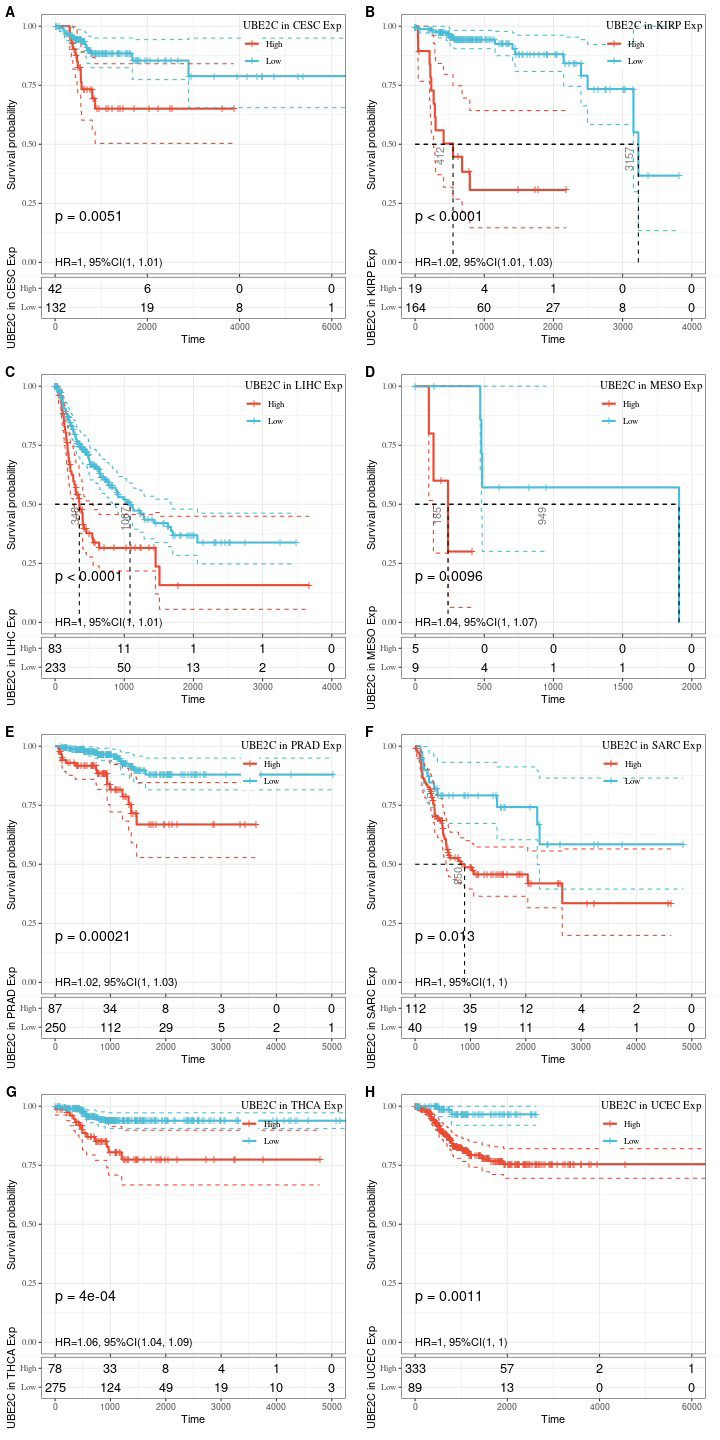

Supplement: Supplementary file 6 [file Image4.jpg]

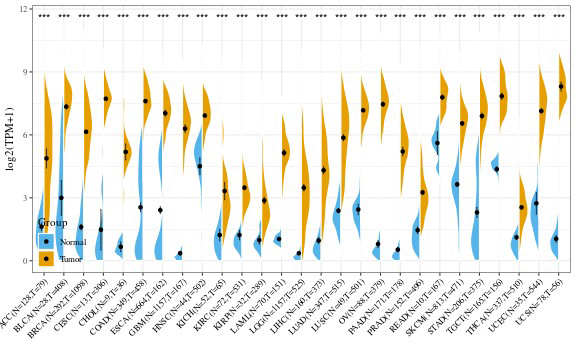

Supplement: Supplementary file 7 [file Image1.jpg]
